# A Positive Selection for Nucleoside Kinases in *E*. *coli*

**DOI:** 10.1371/journal.pone.0162921

**Published:** 2016-09-27

**Authors:** Nirav Y. Shelat, Sidhartha Parhi, Marc Ostermeier

**Affiliations:** 1 Chemical Biology Interface Graduate Program, Johns Hopkins University, 3400 N. Charles St., Baltimore, MD 21218, United States of America; 2 Department of Chemical & Biomolecular Engineering, Johns Hopkins University, 3400 N. Charles St., Baltimore, MD 21218, United States of America; University of Houston, UNITED STATES

## Abstract

Engineering heterologous nucleoside kinases inside *E*. *coli* is a difficult process due to the integral role nucleosides play in cell division and transcription. Nucleoside analogs are used in many kinase screens that depend on cellular metabolization of the analogs. However, metabolic activation of these analogs can be toxic through disruptions of DNA replication and transcription because of the analogs’ structural similarities to native nucleosides. Furthermore, the activity of engineered kinases can be masked by endogenous kinases in the cytoplasm, which leads to more difficulties in assessing target activity. A positive selection method that can discern a heterologous kinases’ enzymatic activity without significantly influencing the cell’s normal metabolic systems would be beneficial. We have developed a means to select for a nucleoside kinase’s activity by transporting the kinase to the periplasmic space of an *E*. *coli* strain that has its PhoA alkaline phosphatase knocked out. Our proof-of-principle studies demonstrate that the herpes simplex virus thymidine kinase (HSV-TK) can be transported to the periplasmic space in functional form by attaching a *tat-*signal sequence to the N-terminus of the protein. HSV-TK phosphorylates the toxic nucleoside analog 3’-azido-3’-deoxythymidine (AZT), and this charged, monophosphate form of AZT cannot cross the inner membrane. The translocation of HSV-TK provides significant resistance to AZT when compared to bacteria lacking a periplasmic HSV-TK. However, resistance decreased dramatically above 40 μg/ml AZT. We propose that this threshold can be used to select for higher activity variants of HSV-TK and other nucleoside kinases in a manner that overcomes the efficiency and localization issues of previous selection schemes. Furthermore, our selection strategy should be a general strategy to select or evaluate nucleoside kinases that phosphorylate nucleosides such as prodrugs that would otherwise be toxic to *E*. *coli*.

## Introduction

Nucleoside kinases are key enzymes in bacterial and mammalian cell reproduction. Because of their involvement in metabolizing nucleosides and maintaining nucleotide pools, nucleoside kinases are major regulators of cellular processes. Nucleoside and nucleotide levels are extensively involved in events such as cell replication, DNA and RNA synthesis, cell signaling and stress response. Since they play such significant roles in essential processes, the last few decades have seen a surge in the development of nucleoside analogs as anticancer and antiviral agents [1]. These nucleoside analogs work by hijacking the machinery of the host cell or virus to inhibit the proper function of processes like DNA or RNA synthesis, eventually leading to the destruction of the host [1]. Anticancer and antiviral nucleoside analogs differ in that antiviral analogs are designed to have lower toxicity towards mammalian systems [1]. Most viruses have their own nucleoside kinases, and the chemical design of antiviral agents is targeted to make these agents substrates of those specific kinases [1,2]. Since most viral nucleoside kinases share little homology towards mammalian or bacterial kinases, the antiviral analogs are not as easily metabolized by eukaryotic or prokaryotic systems. This key difference provides a higher dose tolerance for antiviral agents but also makes their usefulness restricted to viral infections.

However, advances in targeted gene delivery have shown some promise and may provide a way to implement antiviral agents as anticancer agents. If mammalian cells, such as cancer cells, are targeted to express these non-mammalian genes, the cells obtain the ability to activate antiviral prodrugs [3]. This causes toxicity in the targeted mammalian cells as the metabolized antiviral agents cause obstructions in DNA and RNA synthesis [3]. The development of better gene delivery systems has led to a resurgence in the engineering of non-mammalian proteins for gene therapy and medical imaging purposes. Focus has increased in engineering nucleoside kinases, such as the herpes simplex virus thymidine kinase (HSV-TK) (EC 2.7.1.21), with higher activity towards antiviral prodrugs and medical imaging analogs such as 3’-azido-3’-deoxythymidine (AZT) and [^18^F]-2′-fluoro-2′deoxy-1h-D-arabionofuranosyl-5-ethyl-uracil (^18^F-FEAU) [4–7]. However, the paucity of methods for selecting for engineered nucleoside kinases in a high-throughput manner presents a limitation for their engineering.

There are two key factors that make engineering nucleoside kinases difficult. First, selecting for nucleoside kinase activity traditionally involves utilizing a toxic nucleoside analog [4,6,8]. For in vivo selections, phosphorylation of the analog by the engineered kinase results in the toxic effects of the analog’s metabolites, which obstruct DNA and RNA synthesis [9,10]. When the concentration of these metabolized nucleoside analogs are high enough, the resulting obstructions eventually lead to cell death and thereby place strict limitations on the activity windows that can be found for engineered kinases in the selection [11]. Second, it is difficult to separate engineered nucleoside kinase activity from endogenous nucleoside kinase activity for in vivo selections. This adds to the complexity in identifying higher activity variants in direct evolution selections and requires additional screens to be able to clarify the efficacy of an engineered variant [12].

Here, we have developed a bacterial positive selection for nucleoside kinases that addresses both of these issues. The selection involves exporting the HSV-TK to the periplasm of *E*. *coli* BW14012 strain (*phoA*^*–*^). The HSV-TK protein has a tat-signal sequence attached to its N-terminus. After translation and folding of the kinase in the cytoplasm, the *tat-*signaling pathway recognizes the signal sequences and exports the fully-folded protein to the periplasmic space of the bacteria [13]. Upon translocation, periplasmic peptidases cleave the signal sequence off [13]. In the periplasm, HSV-TK retains its kinase activity and is capable of phosphorylating extracellular nucleosides and nucleoside analogs [14]. In the presence of AZT, the kinase phosphorylates the nucleoside analog, thereby preventing the molecule from crossing the inner membrane of the cell. Inside the cell, AZT and AZP-MP would be further metabolized and inhibit DNA and RNA synthesis. Thus, periplasmic kinase activity prevents toxicity and allows the cell to survive in this positive selection.

There are two specific benefits to sending a heterologous kinase to the periplasm of *E*. *coli* for protein engineering. First, by exporting the protein to the periplasm, its activity is spatially segregated from endogenous kinases in the cytoplasm. This helps reduce ambiguity about the source of kinase activity being from the engineered protein or endogenous enzymes. Second, nucleosides and nucleoside analogs that are phosphorylated by the kinase in the periplasmic space cannot breach the inner membrane. In normal cells, the intake of nucleoside analogs, such as the prodrug AZT, is followed by its cytoplasmic phosphorylation via nucleoside kinases [15]. This phosphorylation event instigates the metabolism of the nucleoside analog in to its triphosphate form and eventual incorporation into DNA or RNA, through which it provides its toxicity [12]. The addition of a charged moiety in the periplasm inhibits the nucleoside analog from crossing the inner membrane and thus, the bacterium becomes resistant to the drug. The deletion of the PhoA in *E*. *coli* BW14012 ensures that phosphorylated nucleosides in the periplasm are not dephosphorylated back in to their original, transport competent form [16,17].

## Materials & Methods

### Kits & Reagents

Unless otherwise specified, all molecular biology protocols were performed using NEB’s High-Fidelity Phusion Master Mix for PCR, Invitrogen’s Gel Purification Kit for gel extraction and Zymo’s DNA Clean & Concentrator kit for DNA purification (5 μg loading capacity). Qiagen’s QIAprep Spin Miniprep Kit was used to isolate plasmid DNA from cell culture. 3’-Azido-3’-Deoxythymidine (Azidothymidine, AZT) was purchased from Sigma-Aldrich. 5’-Triphosphate -3’-Azido-3’-Deoxythymidine (AZT-TP) was obtained through US Biological Life Sciences. All nucleotide oligomers and gBlocks were ordered from Integrated DNA Technologies. Ligations were performed using New England Biolabs’ T4 DNA Ligase Buffer (10x) and T4 DNA Ligase (400,000 units/mL).

### Strains

*E*. *coli* strains BW14012 (*F*^*-*^, *Δ(codB-lacI)3*, *ΔphoA532*, *Δ(phnJ-mel)524(Tn5-1/132)*, *[phn*_*B*_*])* and W3110 *(F*,^*-*^ *lambda*^*-*,^ *IN(rrnD-rrnE)1*, *rph-1)* were obtained from the Coli Genetic Stock Center at Yale University. *E*. *coli* 5α *(fhuA2 Δ(argF-lacZ)U169 phoA glnV44 Φ80 Δ(lacZ)M15 gyrA96 recA1 relA1 endA1 thi-1 hsdR17)* was purchased from New England Biolabs.

### pSkunk2-hsvtk Plasmid Construction & active site deletion

The original *hsv-tk* gene was provided by Dr. Margaret Black (University of Washington) in a plasmid dubbed “pMCC”. All experiments were conducted using the pSkunk2 plasmid, which was previously developed in the lab [18]. Primer pairs binding to the beginning of *hsv-tk* and the end of the *hsv-tk* gene in pMCC were designed. The forward primer that overlapped the start codon of *hsvtk* had a flanking NcoI restriction site (5-ATATTAACCATGGATGGCTTCGTACCCCTGCCATC-3’) and the reverse primer that overlapped the stop codon of *hsvtk* had a SalI restriction site (5’-ATATTAACCATGGATGGCTTCGTACCCCTGCCATC-3’). PCR was performed using the pMCC vector as a template with the forward and backward primers. The protocol utilized an initial 2 min 98°C denaturation step, followed by 25 cycles of 98°C denaturation for 30 seconds, 57°C annealing step for 20 seconds, 72°C elongation step for 1 min. There was a final 5 min. 72°C elongation step to finish the amplification. The amplified *hsv-tk* was then gel extracted and concentrated.

pSkunk2 was purified from *E*. *coli* 5α by using a miniprep kit to get isolated plasmid. The low copy plasmid contains a *tac* promoter inducible with isopropyl β-D-1-thiogalactopyranoside (IPTG) and provides streptomycin resistance through an *aadA* gene. Both the isolated plasmid and the amplified *hsv-tk* were then individually subjected to a double restriction digest step. In a 20 μL reaction, 1 μg of plasmid or *hsvtk* was added to NEB’s CutSmart buffer (10x), SalI-HF (NEB), NcoI-HF (NEB) and deionized water. The digestion was run for 1 hour, after which the DNA was purified.

The resulting products of linearized, digested pSkunk2 and *hsvtk* were then ligated together to finish cloning the gene in to the plasmid. The DNA ligation used 100 ng of linearized plasmid with 100 ng of the linearized, digest *hsvtk* gene (approximately 1:3 plasmid to insert ratio) in a 20 μL reaction. The appropriate amounts of T4 DNA Ligase & Buffer were added as recommended by NEB. The reaction was conducted for 1 hour and then purified for DNA. Two μL of the purified DNA ligation was transformed in to *E*. *coli* 5α using electroporation. Colonies from the transformation were sent for sequencing to confirm proper insertion.

Confirmed pSkunk2-*hsvtk* was then subjected to a deletion step using restriction enzymes to yield an inactive *hsvtk* variant. The pMCC plasmid underwent KpnI and SacI digestion, which removed 77 base pairs from the *hsv-tk* gene. A ligation with a small nucleotide sequence with corresponding KpnI and SacI flanks (Forward: 5’-CCCCTCGAGCGCGGTAC-3’, Reverse: 5’-CGCGCTCGAGGGGAGCT-3’) recircularized the plasmid and resulted in an *hsv-tk* with a 66 base pair deletion in its active site. This inactivated form of *hsv-tk* has been used before as a negative control [19]. The inactive *hsv-tk* gene was dubbed “*hsv-tkD*”.

### Addition of signal sequences HSV-TK

Circular polymerase extension cloning (CPEC) was used to add three signal sequences to the front of the start codon of *hsvtk* on pSkunk2. Oligonucleotides targeted to linearize pSkunk2-*hsvtk* from the start codon of the gene were ordered (Forward: 5’-ATGGCTTCGTACCCCTGCCA-3’, Reverse: 5’-CCATGGATCCTTCCTCCTGTGT-3’). PCR was performed to linearize the plasmid, with an annealing step of 57°C annealing step for 20 seconds and 72°C elongation step for 1:50 min. The product was gel extracted and purified. Oligonucleotides containing a signal sequence and flanks homogenous to each end of the linearized plasmid were ordered for all three signal sequences. CPEC was conducted using the linearized DNA fragments according to a previously established protocol [20]. Five μL of CPEC reactions were transformed into chemically competent *E*. *coli* 5α cells (New England Biolabs) and the resulting colonies sequenced for insertion confirmation.

### AZT toxicity assays

pSkunk2-*hsvtk* constructs with the three signal sequences were transformed into *E*. *coli* BW14012. Approximately 2500 CFU (determined in the absence of AZT) of cells harboring each construct were plated on LB Agar plates containing 1 mM isopropyl-β-D-1-thiogalactopyranoside (IPTG), 2.5 mM adenosine triphosphate (ATP), 50 μg/mL streptomycin and 0, 5, 10, 20, 30, 40, 50 or 60 μg/mL AZT. The plates were incubated for 20 hours at 37°C and the percent viability was calculated as the number colonies on each plate divided by the number of colonies on the plate with 0 μg/mL AZT. Assays with *E*. *coli* W3110 and 5α strains were performed in the same manner.

### Western blot of periplasmic and cytoplasmic fractions

*E*. *coli* BW14012 were grown overnight expressing *dsbA-hsvtk* (dsbHSV-TK), *pelB-hsvtk* (pelHSV-TK), *tat-hsvtk* (tatHSV-TK) or inactive *tat-hsvtk* (tatHSV-TKΔ). The optical densities (OD) normalized and the cells subjected to osmotic shock to collect the periplasmic fraction [21]. Cultures were pelleted through centrifugation and then resuspended in cold sucrose buffer (50 mM Tris-HCl (pH 7.4) with 1 mM EDTA and 20% sucrose). Half the culture volume was used for resuspension. The cells were then shaken on ice for 10 minutes and then pelleted again through centrifugation at 4°C. The supernatant was removed and the pelleted cells were resuspended in cold 5 mM MgCl_2_ solution (in a fourth of the culture volume). The resuspended cells were shaken on ice for 15 minutes and centrifuged down again at 4°C. The supernatant contained the periplasmic fraction and was collected. The pelleted cells were then subjected to cell lysis using Novagen’s BugBuster agent to collect the cytoplasmic fraction. The protocol for lysis was conducted as indicated in the kit and soluble fractions were collected.

Periplasmic and cytoplasmic fractions of each variant were electrophoresed on a NuPage 4–12% Bis-Tris protein gel (ThermoFisher Scientific) using SDS-PAGE. The proteins were then transferred on to a polyvinylidene fluoride (PVDF) membrane using Biorad’s Trans-Blot SD Semi-dry Transfer Cell for 15 minutes at 15V. The primary antibody against HSV-TK (Santa Cruz Biotechnologies, Inc.) was incubated with the membrane after protein transfer in Tris-buffered saline-Tween (TBST) buffer at a 3:500 antibody:buffer ratio for 1 hr at 4°C. The anti-goat secondary antibody (Biorad Laboratories, Inc.) was loaded on to the western blot in TBST buffer at the same ratio as the primary antibody. No wash steps or blocking steps were performed. A control to ensure purity of periplasmic fractions was conducted by running the same fractions through the western blot protocol described above using a GroEL antibody (Sigma-Aldrich) as the primary antibody instead of the HSV-TK antibody. A blocking step was conducted before the addition of the primary antibody for 2 hours at 4°C using 2% milk in TBST buffer. The primary antibody:buffer ratio was 1:50000. An anti-rabbit secondary antibody (Biorad Laboratories, Inc.) was used in a 1:500 ratio. We visualized the western blots by adding HRP substrate using Biorad’s Clarity Western ECL Substrate as per the protocol provided. The chemiluminescence was observed using Biorad’s Universal Hood II and Quantity One Software.

### Selection enrichment assay

BW14012 cells harboring wild-type *hsv-tk (tat)* were mixed with cells holding *hsvtkΔ (tat)* in a 1:10000 ratio. A total of 500,000 CFU of the mixture was plated on large bioassay plates containing LB Agar, IPTG (1 mM), ATP (2.5 mM), streptomycin (50 μg/mL) and AZT (40 μg/mL). The plates were incubated for 24 hours at 37°C. Colonies were subjected to a colony screening PCR to determine which form of *hsvtk* they harbored. There was a 66 bp size difference between wild-type *hsv-tk* and the smaller, inactive *hsv-tk*. The PCR protocol was identical to the one described earlier for *hsvtk* amplification and the same primers were used. After analysis of DNA size by agarose gel electrophoresis, the plasmids of five colonies were sequenced. The minimum enrichment rate was found by dividing the ratio of HSV-TK positive colonies to total colonies by the mix ratio (1:10,000).

## Results

**Fig 1:**
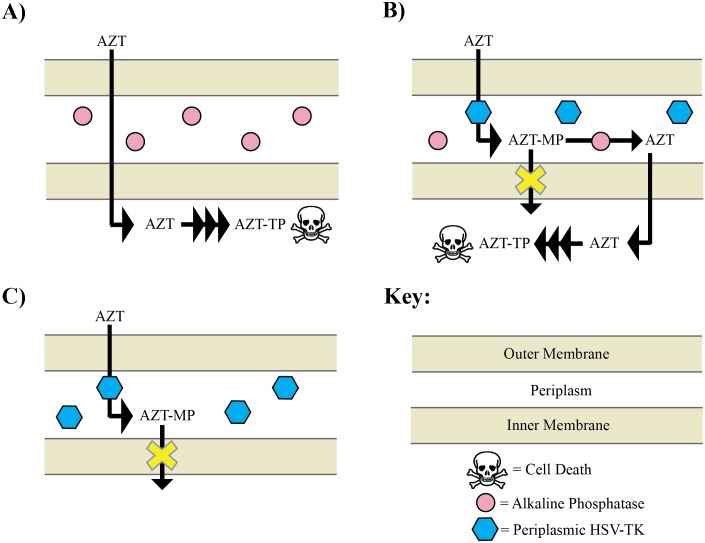
Positive selection for HSV-TK activation of AZT. A) In typical cells, AZT penetrates the outer membrane, periplasm and inner membrane to be metabolized in to its triphosphate form to generate genotoxicity towards *E*. *coli*. B) When HSV-TK is exported to the periplasm AZT is phosphorylated in the periplasm; however, PhoA counteracts by dephosphorylating the AZT-MP, allowing AZT to penetrate the inner membrane causing genotoxicity. C) In cells lacking PhoA but containing periplasmic HSV-TK, AZT is phosphorylated and AZT-MP cannot cross the inner membrane. Thus, periplasmic HSV-TK provides AZT resistance to *phoA*^–^strains.

We used HSV-TK as the kinase and AZT as the prodrug (Fig 1) in our proof-of principle studies. We first sought to test the underlying assumptions that phosphorylated AZT cannot breach the inner membrane and that phosphorylated AZT monophosphate (AZT-MP) is much less toxic than AZT when supplied in the growth media. Since AZT-MP is not available commercially, we substituted AZT triphosphate (AZT-TP) to act as a proxy. Bacterial outer membranes allow small molecules to pass through using either a lipid-mediated pathway or through passive diffusion of hydrophilic molecules up to 600 Daltons using porins [13,22]. We presumed that AZT (267 Daltons) and AZT-TP (507 Daltons) utilize the latter. We challenged *E*. *coli* DH5α cells to grow on solid media containing AZT or AZT-TP. Whereas AZT prevents growth at just 0.5 μg/ml, AZT-TP could be present up to at least 10 μg/ml media without any decrease in cell viability. This is consistent with the notion that the phosphorylated molecule is not toxic to cells because it cannot penetrate the inner membrane.

### Periplasmic transport of HSV-TK

**Fig 2:**
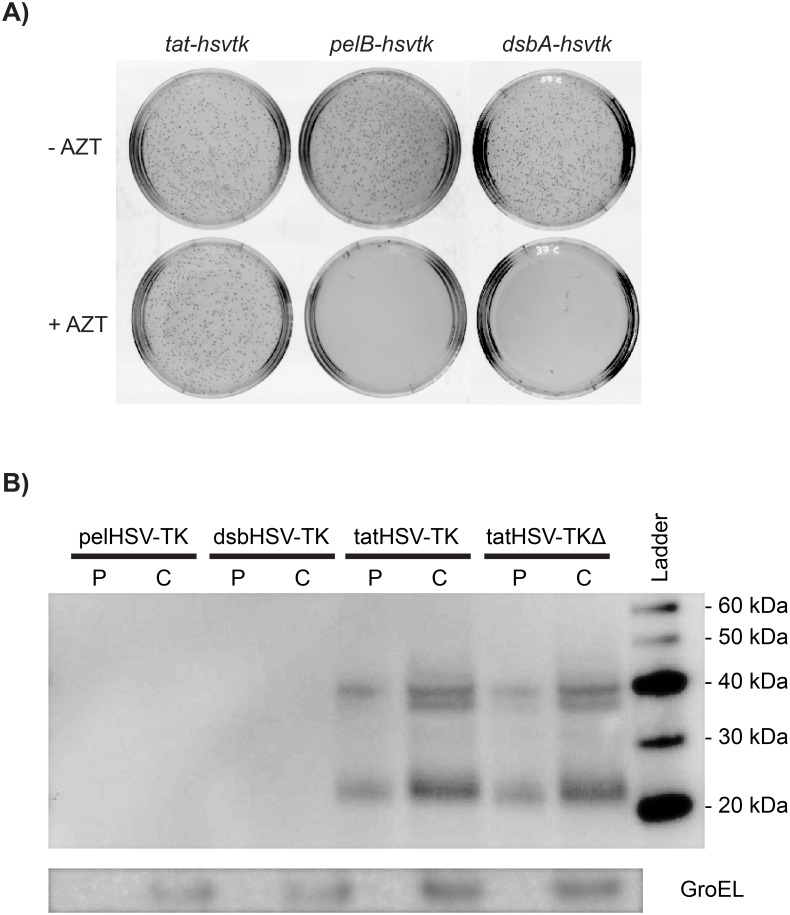
Export of active HSV-TK using the tat signal sequence. A) 2500 CFUs of *E*. *coli* BW14012 cells with *tat-hsvtk*, *pelB-hsvtk or dsbA-hsvtk* were plated on LB agar plates containing 50 μg/mL streptomycin, 2.5 mM ATP, 1mM IPTG, and the presence (bottom row) or absence (top row) of 10 μg/mL AZT. The plates were incubated for 20 hours at 37°C. B) Western blot using anti-HSV-TK antibodies of periplasmic (“P”) and cytoplasmic (“C”) fractions of cells expressing the indicated proteins. The expect size of HSV-TK is 41 kDa. A control using anti-GroEL antibodies was used to confirm there was no cytoplasmic protein contamination in the periplasmic fractions.

For the selection to work properly, HSV-TK must be exported to the periplasm in functional form. Previous studies have confirmed the presence of HSV-TK in the periplasm through western blot analysis of isolated, soluble periplasmic fractions [11]. The study utilized a *pelB* signal sequence to export HSV-TK to the periplasm in *Salmonella typhimurium* but did not test other export signal sequences. We expected that efficient transport of HSV-TK into the periplasm would generate stronger resistance towards AZT. To identify the most efficient methods of transport *E*. *coli*, we tested three separate periplasmic-export signal sequences. The *pelB* and *dsbA* signal sequences export unfolded protein into the periplasmic space using the Sec pathway and SRP pathway, respectively [23]. The *tat* signal sequence sends folded proteins into the periplasm using the twin-arginine pathway [24]. We fused the wild-type *hsv-tk* gene to the *dsbA*, *pelB*, and *tat* export signal sequences on the pSkunk2 plasmid and transformed into the *phoA*^–^*E*. *coli* strain BW14012 [18]. Cells harboring each construct were plated on solid media containing 2.5 mM ATP and different concentrations of AZT. We added ATP because HSV-TK transfers the phosphate from ATP, and ATP is not naturally present in the periplasm. The *tat* signal sequence provided robust resistance even at 10 μg/ml AZT (Fig 2a). The *pelB* and *dsbA* signal sequences provided no resistance, as no growth was observed even at 1 μg/ml AZT. In contrast, high levels of AZT resistance (up to 40 μg/mL) were observed for cells containing wild-type HSV-TK with a *tat-*signal sequence (tatHSV-TK). Export of a mutated, inactive version of HSV-TK (tatHSV-TKΔ) via the Tat pathway provided no AZT resistance. This inactive, mutated version of HSV-TK is previously described [19] and contains a deletion in the active site of the enzyme.

To confirm that HSV-TK was being successfully transported into the periplasm, we performed a western blot on the periplasmic and cytoplasmic fractions of *E*. *coli* BW14012 expressing tatHSV-TK, dsbHSV-TK or pelHSV-TK (Fig 2b). A catalytically inactive HSV-TK control with a tat signal sequence, tatHSV-TKΔ, was also tested for proper transport. Both HSV-TKs with the tat signal were present in the cytoplasmic and periplasmic fractions but HSV-TK conjugated to the pelB or dsbA signal sequences were not detected in either fraction. These results indicate the necessity for folded protein translocation for HSV-TK in order to provide AZT resistance.

### Knock out of PhoA activity is essential for the positive selection

In the preceding experiment, we used a strain of *E*. *coli* deficient in the periplasmic phosphatase PhoA. In theory, PhoA could remove phosphate groups from AZT that had been phosphorylated by HSV-TK. This would allow the molecules to pass through the inner membrane through nucleoside transporters and prevent the cell from possessing strong resistance to nucleoside analogs [25]. We tested the importance of using a *phoA*^–^strain for the positive selection by comparing it to strains with a functional PhoA. We tested the AZT sensitivity of cells expressing wild-type and inactive HSV-TKD (both fused to Tat signal sequences) in the *phoA*^+^ strains 5α and W3110. Strains 5α and W3110 showed much less resistance than the *phoA*^–^BW14012 strain. Expression of tatHSV-TK in 5α and W3110 provided only marginal resistance to AZT. Only 16% and 18%, respectively, of the cells plated were able to grow in the presence of 1 μg/mL AZT, and no colonies for either strain formed on plates containing 10 μg/ml AZT. In contrast, BW14012 cells expressing tatHSV-TK showed no growth defect at 10 μg/ml AZT and had 65% viability even at 40 μg/ml. We postulate that the substantial toxicity of AZT in 5α and W3110 is due to PhoA dephosphorylating AZT-MP, allowing it to enter the cell. Although 5α and W3110 are not isogenic to BW14012, we believe it is likely that the lack of PhoA in *E*. *coli* BW14012 is a crucial component of the positive selection

### AZT toxicity assays

**Fig 3:**
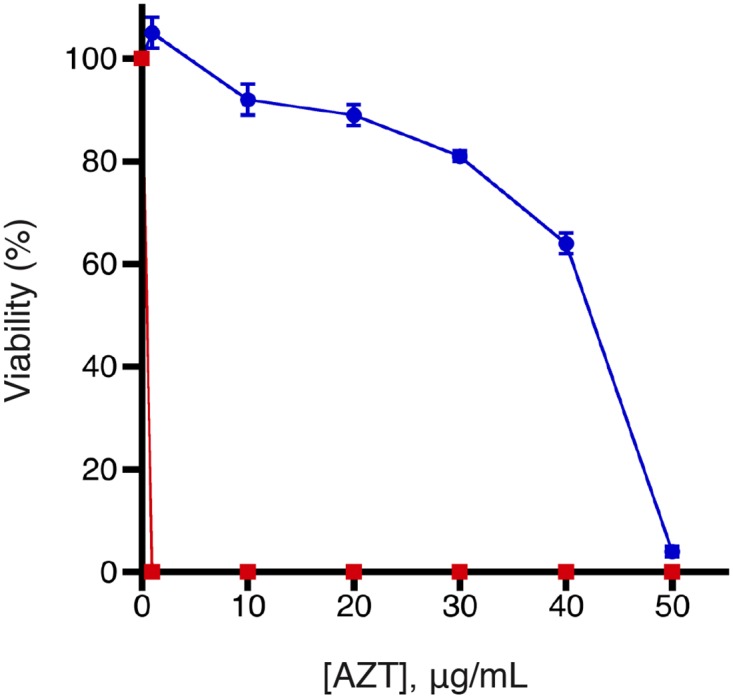
Viability of *E*. *coli* BW14012 cells expressing tatHSV-TK (blue circles) or tatHSV-TKD (red squares) as a function of AZT concentrations. Cell viability is expressed relative to the number of colonies at 0 μg/mL AZT for each strain type.

By comparing the magnitude of AZT resistance in the presence of periplasmic kinase and an inactivated counterpart, it was possible to determine the optimal conditions for a positive selection. We quantified cell viability on solid media as a function of AZT concentration for cells expressing tatHSV-TK and cells expressing the inactive tatHSV-TKΔ, both in *E*. *coli* BW14012 (Fig 3). Cells with tatHSV-TK showed cell viability of 65 ± 2% at 40 μg/mL AZT while those with tatHSV-TKΔ had <0.01% viability even at 1 μg/mL AZT (Fig 3). At concentrations up to 20 μg/mL AZT, almost full (>95%) viability was observed with cells expressing tatHSV-TK. After 20 μg/mL AZT, there was a decline in cell viability until 40 μg/mL AZT. The cells exhibited a drastic reduction in viability beyond this concentration of AZT. This drop represents a “resistance threshold” that wildtype HSV-TK kinase activity cannot overcome, but one that an improved HSV-TK might.

### Demonstration of the selection

**Fig 4:**
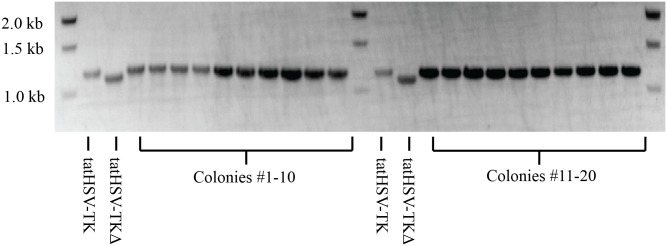
PCR assay demonstrating at least a 10,000-fold enrichment for active tatHSV-TK over an inactive tatHSV-TKΔ. BW14012 cells expressing one of the two proteins were mixed at a 1:10,000 ratio (tatHSV-TK:tatHSV-TKD) ratio. Twenty colonies formed when 500,000 CFUs (determined under non-selective conditions) were plated on 40 μg/ml AZT. PCR-amplified *hsvtk* genes from these 20 colonies indicated that all twenty contained tatHSV-TK. Expected band sizes of tatHSV-TK and tatHSV-TKD in this PCR assay are 1226 bp and 1160 bp, respectively.

We expect that the drastic drop in viability in *E*. *coli* BW14012 with periplasmic HSV-TK around 40 μg/mL AZT will allow for a positive selection that can identify engineered HSV-TK with increased kinase activity. We performed a mock selection experiment to test how effectively our positive selection can identify high activity kinases over kinases with less activity. The mock selection involved plating mixtures of BW14012 cells expressing tatHSV-TK or tatHSV-TKΔ (1:10,000 ratio) on solid media containing 40 μg/mL AZT. We plated 500,000 CFUs on solid media and 20 colonies formed. With a 1:10,000 mix ratio and a 65% observed viability for cells expressing tatHSV-TK at this AZT concentration, we expected approximately 33 colonies. Colony PCR was conducted to identify whether these colonies harbored tatHSV-TK or tatHSV-TKD. All 20 colonies had the wild-type HSV-TK (Fig 4). This corresponds to at least a 10,000-fold enrichment for AZT kinase activity.

## Discussion

This study establishes a new method for positive selection of nucleoside kinase activity in *E*. *coli*. The selection is capable of spatially separating the heterologous kinase’s activity from endogenous kinases. The translocation of the kinase to the periplasm provides a way to test phosphorylation activity without interference from endogenous kinase activity and without disrupting internal cellular function. In the presence of periplasmic HSV-TK, an 80-fold increase in resistance towards AZT was observed when compared to inactive HSV-TK. This resistance sharply declined beyond 40 μg/mL AZT. We propose that this viability threshold provides an opportunity to select for higher activity variants of engineered HSV-TK in directed evolution experiments. The high degree of enrichment seen during mock selections at 40 μg/ml AZT supports this proposal. However, the ability of the selection to distinguish HSV-TK variants with small improvements in activity has not been tested.

While HSV-TK is known for its high activity towards nucleoside analogs, the robustness of the selection should also permit less promiscuous nucleoside kinases, such as deoxycytidine kinase, to generate resistance towards other nucleoside analogs [26]. In theory, this selection could be applicable to any nucleoside kinase that can be functionally expressed in the periplasm and can phosphorylate toxic nucleoside analogs. Additionally, a similar positive selection process can be envisioned for nucleoside phosphatases in which periplasmically-expressed nucleoside phosphatases would remove phosphates from essential nucleotides to allow them to be transported in the cytoplasm in nutrient deficient conditions. This would rescue the cell from nucleoside starvation.

The selection strategy should work for kinase activity on any nucleoside that is toxic to *E*. *coli*. To apply this selection to a nucleoside kinases one must first establish that the nucleoside is toxic to *E*. *coli*, which requires that it be transported into the cytoplasm. The outer membrane of *E*. *coli* is permeable to a vast variety of molecules through passive diffusion from outer membrane porins but its selectivity substantially increases at the inner membrane. Uncharged nucleosides are transported to the cytoplasm via *nupC* and *nupG* [27]. These two nucleoside permeases have also displayed the ability to translocate a number of nucleoside analogs [25,27]. After transport into the cytoplasm, the nucleoside must be toxic itself or metabolized into toxic compounds. This process could be facilitated by heterologously expressed nucleoside kinases, should endogenous, cytoplasmic kinases be unable to do this reaction.

The selection also requires that the nucleoside kinase to be subject to selection is capable of being exported to the periplasm in functional form. For HSV-TK, we found that the Tat pathway for periplasmic translocation of folded proteins performed best, but other kinases may work better with one of the other export pathways. Some nucleoside and nucleoside analogs conceivably could interact with proteins in the periplasm and this interaction may affect the ability of the kinase to phosphorylate the nucleoside to provide resistance. However, this is a low-probability event since nucleosides have no known function outside the cytoplasm in bacteria. Although *E*. *coli* scavenges inorganic phosphate from nucleotides using PhoA, it has been suggested that other putative phosphatases may also be able to fulfill this role under highly stressed conditions [28,29]. In such a scenario, these genes must also be knocked out for the selection to work properly. This scenario was not observed under the stress of AZT and would not be expected for other nucleoside analogs; scavenging phosphatases are regulated by the abundance of extracellular inorganic phosphates, a molecule that the selection media must provide in order for any engineered kinase to work [29].

The key advantage of the selection scheme in this study is in the kinase’s localization. Previous positive selection methods have attempted to dampen the noise from endogenous enzymes and reduce disruptions to cell function by genetically knocking out certain bacterial kinases or inhibiting enzymes involved in nucleoside metabolism with small molecules [4,30–32]. These methods yielded a positive selection, but one with strict limitations. The selections were stifled by the use of the small molecule protein inhibitors, which can have some base-line toxicity, and perturbations to normal bacterial metabolism through gene deletions. In addition to the base toxicity, some inhibitors used to maintain selection fidelity were partially metabolized by the engineered enzyme [33]. This resulted in a feedback loop that placed a cap on the level of kinase activity that could be selected. Engineered kinases with improved activity could metabolize the inhibitor at a higher rate, which would begin generating more toxicity. These factors convolute directed evolution experiments by increasing false positive rates and potentially killing the most effective variants. Most positive selections in these experiments needed multiple rounds of screening after selections to identify successfully engineered enzymes amongst the false positives. By localizing the kinase to the periplasm, the need to inhibit endogenous kinases through gene deletions and molecular inhibitors has been removed. The only required deletion is for phoA, a periplasmic enzyme that is not involved in metabolism inside the cell. This yields a selection that is free of metabolic stress from protein inhibitors and gene deletions.

We believe that the positive selection developed in this proof-of-principle study should be capable of identifying engineered nucleoside kinases and possibly nucleoside phosphatases with increased specific activity or expression levels compared to wild-type. Discovery of more substrate-specific, highly stable and highly active variants of such enzymes has direct implications for biotechnology. These modified enzymes can also be used as reporter genes for experiments or potentially be utilized in GDEPT experiments. Further work will need to be done to examine if this selection has a bias towards selecting for stability (i.e. increased protein abundance) or specific catalytic activity of kinases.
